# Personalized treatment for hepatocellular carcinoma in the era of targeted medicine and bioengineering

**DOI:** 10.3389/fphar.2023.1150151

**Published:** 2023-05-05

**Authors:** Hang Sun, Huayu Yang, Yilei Mao

**Affiliations:** Department of Liver Surgery, Peking Union Medical College (PUMC) Hospital, Peking Union Medical College (PUMC) and Chinese Academy of Medical Sciences (CAMS), Beijing, China

**Keywords:** hepatocellular carcinoma, target therapy, personalized therapy, bioengineering technolog, precision medicine

## Abstract

Hepatocellular carcinoma (HCC) is a major global health burden, causing approximately 8.3 million deaths each year, and it is the third leading cause of cancer-related death worldwide, with a relative 5-year survival rate of around 18%. Due to the advanced stage of diagnosis in most patients, systemic treatment based on targeted therapy has become the only feasible option. Genomic studies have established a profile of molecular alterations in hepatocellular carcinoma with potentially actionable mutations, but these mutations have yet to be translated into clinical practice. The first targeted drug approved for systemic treatment of patients with advanced hepatocellular carcinoma was Sorafenib, which was a milestone. Subsequent clinical trials have identified multiple tyrosine kinase inhibitors, such as Lenvatinib, Cabozantinib, and Regorafenib, for the treatment of hepatocellular carcinoma, with survival benefits for the patient. Ongoing systemic therapy studies and trials include various immune-based combination therapies, with some early results showing promise and potential for new therapy plans. Systemic therapy for hepatocellular carcinoma is complicated by the significant heterogeneity of the disease and its propensity for developing drug resistance. Therefore, it is essential to choose a better, individualized treatment plan to benefit patients. Preclinical models capable of preserving *in vivo* tumor characteristics are urgently needed to circumvent heterogeneity and overcome drug resistance. In this review, we summarize current approaches to targeted therapy for HCC patients and the establishment of several patient-derived preclinical models of hepatocellular carcinoma. We also discuss the challenges and opportunities of targeted therapy for hepatocellular carcinoma and how to achieve personalized treatment with the continuous development of targeted therapies and bioengineering technologies.

## 1 Introduction

Worldwide, hepatocellular carcinoma (HCC) remains the fourth most common cause of cancer-related death, and its global burden continues to increase each year (Villanueva, 2019; Llovet et al., 2021; Vogel et al., 2022). With the development of diagnosis and treatment technologies in recent decades, the treatment of HCC has progressed greatly. However, except for cases detected early, most patients are currently diagnosed at a later stage and curative treatments are frequently not available (Benson et al., 2021; Llovet et al., 2021; Vogel et al., 2022). Therefore, systemic therapies (combination therapies) are the key to the survival of advanced HCC patients (Gordan et al., 2020). Targeted therapy and immunotherapy are the most studied and applied systemic treatment methods in recent years, and they are playing an increasingly important part in the treatment of patients with advanced HCC (Llovet et al., 2018; Huang et al., 2020). In order to enhance the survival rate of HCC patients, precise and individualized treatments will become the future of HCC systemic treatment.

The extensive intratumoral heterogeneity of HCC and the non-negligible drug resistance of targeted drugs are the main obstacles for developing individualized HCC treatments (Fisher et al., 2013; Schulze et al., 2015; Zucman-Rossi et al., 2015; Mcgranahan and Swanton, 2017). In the past, traditional tumor models could not reflect the heterogeneity of different HCC patients nor could they be used for research on targeted drug resistance in different patients hindering the development of personalized treatment for HCC. With the development of bioengineering techniques in recent years, patient-derived liver cancer preclinical models reflecting the complex characteristics of tumors can now be created, showing great promise to benefit the development of personalized medicine for HCC patients and improve clinical outcomes (Bresnahan et al., 2020).

In this review, we examine recent advances in targeted therapies for liver cancer and discuss the application of bioengineered models of liver cancer to personalized treatment of liver cancer, including the novel clinical trials and technology platforms expected to facilitate substantial progress over the next decade.

## 2 Targeted therapy

Targeted therapy embodies the precise treatment for HCC. With the completion of the Genome Project, the molecular alteration profile of HCC is well known (Craig et al., 2020; Rebouissou and Nault, 2020). Numerous studies demonstrated genes from multiple signaling pathways, such as Wnt/β-catenin, P53/cell cycle regulation, oxidative stress, epigenetic modifiers, et al., were frequently mutated in HCC (Boyault et al., 2007; Hoshida et al., 2009; Schulze et al., 2015). Molecularly targeted drugs modulating these molecules and pathways have become a hot area in liver cancer research, but only a small number of tumors, about 25%, have potentially targetable drivers (Schulze et al., 2015). Therefore, it is extremely difficult to develop effective therapies other than surgery for HCC. Similarly, various cytokines involved in these signaling pathways, such as vascular endothelial growth factor (VEGF), fibroblast growth factor (FGF), transforming growth factor-α (TGF-α), and insulin-like growth factor-II have also been extensively studied. Among them, anti-angiogenic drugs targeting the VEGF play an important role in the targeted therapy of HCC (Weis and Cheresh, 2011).

### 2.1 Sorafenib

The groundbreaking study (SHARP) in 2007 demonstrated that the tyrosine kinase inhibitor (TKI) sorafenib resulted in a 3-month overall survival (OS) benefit to patients with advanced liver cancer (Llovet et al., 2008; 2018). Subsequent clinical trials also proved the effectiveness of sorafenib in improving OS and objective response rate (ORR). Sorafenib was the only therapy with confirmed survival benefits for patients with advanced liver cancer for a long time thereafter (Cheng et al., 2009). Although the efficacy and safety of sorafenib have brought improvements to the treatment of HCC, (Bruix et al., 2012; Raoul et al., 2012; EASL Clinical Practice Guidelines and European Association for the Study of the Liver, 2018), the average survival time of patients is still less than 1 year. Therefore, some studies have turned their attention to combination therapy. The existing clinical trials proved that in the treatment of advanced HCC, sorafenib combined with Transarterial chemoembolization (TACE), external irradiation, and other therapies prolong the disease-free survival time and OS time compared with sorafenib alone (Qu et al., 2012; Meyer et al., 2017; Zhao et al., 2019). In recent years, targeted therapies have continued to develop. Currently, there are first-line TKIs lenvatinib and donafenib, as well as second-line treatments regorafenib, cabozantinib and apatinib, which have been proven to be effective for advanced liver cancer (Table 1).

**TABLE 1:** Approved clinical trials of targeted therapy for advanced HCC.

Name/identifier	Study type	Drug	Target	Line
SHARP/NCT00105443	Phase III	Sorafenib	Multikinases	First-line
REFLECT/NCT01761266	Phase III	Lenvatinib	Multikinases	First-line
RESORCE/NCT01774344	Phase III	Regorafenib	Multikinases	Second-line
CELESTIAL/NCT01908426	Phase III	Cabozantinib	Multikinases	Second-line
REACH-2/NCT02435433	Phase III	Ramucirumab	VEGFR2	Second-line
AHELP/NCT02329860	Phase III	Apatinib	Multikinases	Second-line

### 2.2 Lenvatinib

Compared with sorafenib, lenvatinib has advantages in reducing drug resistance. Drug resistance caused by long-term sorafenib treatment of liver cancer is one of the leading clinical problems at present. Sorafenib resistance is generally believed to be due to the presence of FGF, a pro-angiogenic factor thought to increase tumor cell resistance to anti-VEGF therapy (Tang et al., 2020). As a TKI, lenvatinib can selectively inhibit VEGF receptors (VEGFR) 1-3, FGF receptors 1-4, platelet-derived growth factor receptor-α, RET, and cKIT, which may reduce the occurrence of drug resistance during treatment (Al-Salama et al., 2019). The results of the REFLECT trial in 2018 showed that in the treatment of advanced HCC, the inhibitory effect of lenvatinib on tumors was not inferior to that of sorafenib (Kudo et al., 2018). Lenvatinib especially improved the efficacy of secondary endpoints, compared with sorafenib and it significantly prolonged the median progression-free time and improved the ORR. The curative effect of lenvatinib on patients with hepatitis B virus-related HCC is better than that of sorafenib (Al-Salama et al., 2019). Lenvatinib is the second first-line drug for advanced HCC and was developed 10 years after sorafenib (Hiraoka et al., 2019). It not only provides new drug options for patients with advanced liver cancer but also provides evidence for subsequent drug development.

### 2.3 Regorafenib

The current second-line molecular targeted drugs, regorafenib and cabozantinib, can be used as monotherapy for HCC patients who have progressed on sorafenib. Regorafenib was approved as the first second-line treatment for HCC patients who progressed during or after sorafenib treatment and ushered in the era of second-line and sequential therapy (Finn et al., 2018). Regorafenib is a multi-target TKI discovered during the process of adjusting the molecular structure of sorafenib to optimize its curative effect. It has a stronger effect antagonizing VEGFR kinase and inhibiting TIE2, cKIT, and RET kinases. The results of the RESORCE trial showed that for HCC patients whose tumors continued to progress during sorafenib monotherapy, regorafenib could significantly prolong the patient’s OS (2.8 months), progression-free survival (PFS), and time to progression (TTP) which were significantly longer than those of the placebo group (Bruix et al., 2017). Subsequent relevant clinical studies have also confirmed the effectiveness of regorafenib, and sequential treatment with sorafenib has been shown to be effective for patients with HCC recurrence after liver transplantation (Iavarone et al., 2019; Yoo et al., 2019).

### 2.4 Cabozantinib

Cabozantinib is a TKI targeting kinases such as MET, AXL, and VEGFR1-3. It is also used as a second-line drug for advanced liver cancer that is not responsive to sorafenib treatment. The CELESTIAL Phase 3 clinical trials showed that cabozantinib can significantly prolong the OS of patients (2.2 months) and the ORR and PFS were not significantly different from the RESORCE trial of regorafenib (Abou-Alfa et al., 2018). Cabozantinib, which differs from regorafenib, can be used in patients who are intolerant to sorafenib. However, cabozantinib treatment may have more toxic side effects than regorafenib (Kudo, 2018). The results of the cost-effectiveness analysis also showed that its economic cost is higher than that of regorafenib, although sorafenib-resistant HCC patients can benefit from cabozantinib treatment (Parikh et al., 2017; Soto-Perez-De-Celis et al., 2019). Therefore, choosing the right patient population is very important.

### 2.5 Ramucirumab

Unlike the above-mentioned second-line TKI drugs, ramucirumab is a recombinant monoclonal antibody targeting VEGFR2, which can block the ligand-receptor interaction and its downstream signaling to exert anti-tumor effects (Syed, 2020). Ramucirumab failed to meet its primary endpoint as second-line therapy in the REACH trial and ramucirumab did not significantly improve OS compared with placebo (9.2 months vs. 7.6 months) (Zhu et al., 2015). However, subgroup analysis confirmed that patients with elevated AFP (≥400 ng/mL) could obtain a better survival benefit from ramucirumab treatment (Chau et al., 2017; Zhu et al., 2017; Gilabert and Raoul, 2018). This was subsequently confirmed by the REACH-2 trial, (Zhu et al., 2019), and ramucirumab was the first FDA-approved drug for HCC patients in a biomarker-selected group (AFP ≥400 ng/mL), but its underlying biomarker-driven mechanism still needs to be further explored (Montal et al., 2019; Zhu et al., 2021).

### 2.6 Immune checkpoint inhibitors

In addition to molecularly targeted therapy, immunotherapy is becoming another clinical weapon for the systemic treatment of liver cancer. Immune evasion in HCC is an important cause of tumor progression. Immune checkpoint proteins are glycoproteins on the cell surface that transmit inhibitory signals to T cells and natural killer cells, and are widely expressed on tumor cells, macrophages, dendritic cells, and other immune cells. These proteins inhibit the excessively activated T cell response, thereby acting on the tumor-immune tolerance process. Tumor cells can inhibit the activation of T cells by expressing immune checkpoint-related molecules and escape the body’s immune surveillance (Llovet et al., 2022). In HCC, anti-PD-1 monoclonal antibodies and anti-PD-L1 monoclonal antibodies are currently the most studied, clinically meaningful immune checkpoint inhibitors (ICIs).

As more clinical trials are completed, the number of first- or second-line drugs for immunotherapy also increases. Currently, immune drugs approved for the first-line treatment include atezolizumab, sindilizumab, camrelizumab, and pembrolizumab. The combination of these drugs or with anti-angiogenic molecularly targeted drugs, for example, the combination of atezolizumab and bevacizumab, (Finn et al., 2020), sintilizumab and bevacizumab biosimilar IBI305, (Ren et al., 2021), camrelizumab Mab plus apatinib, (Xu et al., 2021), or pembrolizumab plus lenvatinib, (Ikeda et al., 2019), were promising in completed clinical trials.

The immune drugs that have been approved for second-line treatment include camrelizumab, pembrolizumab, nivolumab, and ipilimumab. Treatment options include immune monotherapy, immunotherapy combined with anti-angiogenic targeted therapy, and combined immunotherapies (El-Khoueiry et al., 2017; Qin et al., 2020; Qin et al., 2022). Overall, combination therapy including immunotherapy significantly improved ORRs and prolonged OS compared with immunotherapy alone (Table 2).

**TABLE 2:** Clinical trials of combination therapy for advanced HCC.

Name/identifier	Study type	Drug	Target	Primary endpoint	Line
IMbrave150/NCT03434379	Phase III	Atezolizumab Bevacizumab	PD-L1	19.2 months (mOS)	First-line treatment
			VEGF	29.8 (ORR)	
NCT03794440	Phase II/III	Sintilizumab IBI305	PD-1	—	—
			VEGF		
RESCUE/NCT03463876	Phase II	Camrelizumab Apatinib	PD-1	—	—
NCT03764293	Phase III		VEGFR2	—	—
KEYNOTE524/NCT03006926	Phase Ib	Lenvatinib Pembrolizumab	Multikinases	9.3 months (mPFS)	—
			PD-1	22 months (mOS)	
				46% (ORR)	
LEAP-002/NCT03713593	Phase III			—	—

## 3 Challenges and opportunities of targeted therapy for HCC

### 3.1 Challenges

As mentioned above, the occurrence and development of HCC is a complex multi-pathway-mediated process. The emergence of the aforementioned targeted drugs has given people more confidence in the future treatment of HCC. New drugs for targeted therapy are also being continuously developed.

Because of the complexity of HCC, monotherapy often leads to dose- or time-dependent severe adverse events (AEs), resulting in treatment interruption due to intolerance. Consequently, the efficacy of single drugs such as TKI or ICI has reached a bottleneck at an OS of 14–16 months. This also suggests that the developmental process of targeted drugs should be changed. Therefore, in the past 2 years, various combinations of ICI and anti-VEGF monoclonal antibodies have been the research focus of HCC-targeted therapy, which has greatly improved the survival rate of advanced HCC patients and created a new combination for targeted therapy (Ouyang et al., 2022).

As mentioned above, for the first-line immunotherapy regimen, the combination of ICIs and anti-VEGF monoclonal antibodies (atezolizumab combined with bevacizumab), has better clinical outcomes than sorafenib and there is no significant difference in the risk of AEs (Finn et al., 2020). The phase II study (RESCUE) of the novel VEGFR2 TKI apatinib in combination with camrelizumab showed an exciting survival benefit and safety profile. An ongoing randomized, open-label, multicenter, phase III trial (NCT03764293) comparing this combination with sorafenib in advanced HCC is promising; however, combination did not meet the expected endpoint. The double-blind, randomized controlled phase III LEAP-002 trial of lenvatinib plus placebo, which completed recently, showed the median OS period of lenvatinib and pembrolizumab was 21.2 months but did not meet its prespecified co-endpoints of significantly improved OS or PFS (Finn Rs, 2022). In conclusion, various combinations of ICIs and anti-angiogenic drugs significantly improved the clinical survival of advanced HCC patients. The clinical applicability of combined targeting is promising, but at the same time, the accompanying risk of AEs cannot be ignored.

### 3.2 Opportunities

The continuous emergence of the above-mentioned targeted drugs and combined treatment options in recent years increased confidence in the treatment of HCC. Nevertheless, it cannot be ignored that HCC is a type of solid tumor with a complex tumor microenvironment consisting of various liver non-parenchymal cells, extracellular matrix proteins, and signaling molecules, which play an important role in tumor evolution and response to treatment by inducing inflammation, angiogenesis, hypoxia, and fibrosis. Drug resistance in HCC is closely related to its tumor heterogeneity and evolution, and drug resistance is also the main reason for targeted therapy treatment failure. Therefore, in the individualized treatment of HCC, avoiding tumor heterogeneity and mastering tumor evolution to overcome drug resistance are the key points to achieving breakthrough progress. In short, identifying specific patient populations that respond to individual treatments and finding clear drug-sensitivity markers are clinical problems that urgently need to be solved for precise targeted therapy.

The rapid development of next-generation sequencing technology (NGS) provides new hope for the precision of targeted therapy (Collins and Varmus, 2015). Biomarker-driven targeted therapy can be adjusted and customized individually by NGS. Therefore, NGS can improve the ability to differentiate individual characteristics of tumors and has the potential to identify new therapeutic targets, thereby ushering in the era of precision medicine (Collins and Varmus, 2015; Karlovich and Williams, 2019). But much remains to be done to successfully bring NGS closer to impacting clinical care in HCC.

Several studies have shown the value of NGS in precisely targeted therapy of HCC. Utilizing archived tumor tissue and baseline plasma samples from HCC patients in the RESORCE trial of regorafenib, a plasma miRNA panel and gene mutation signature in tumors were found to predict response to regorafenib (Teufel et al., 2019). In BIOSTORM, STORM’s biomarker companion study identified polygenic signatures related to improved relapse-free survival (RFS) with sorafenib adjuvant therapy after hepatectomy, which could be used to guide treatment options in the future (Pinyol et al., 2019). In addition, NGS is also a key tool for developing potential drug-sensitivity markers for targeted therapies. Using the FDA-approved 468-genome MSK-IMPACT, Harding et al (Harding et al., 2019) were able to prospectively identify mutations that were predictive of adverse outcomes with sorafenib and ICIs.

Important advances have been made in lung and colorectal cancers by selecting patients for treatment based on their molecular characteristics, whereas HCC is limited by complex significant molecular heterogeneity. But the improved resolution of NGS technology enables the discovery of significant tumor heterogeneity. With an appropriate and well-designed protocol, the prediction of a potential biomarker response to a specifically targeted drug is greatly increased. In recent years, the field of artificial intelligence (AI) has experienced rapid growth, driven by the development of big data models and deep learning algorithms. This technology has shown great potential for predicting targeted therapy biomarkers and managing the prognosis of hepatocellular carcinoma (Chen et al., 2020; Ahn et al., 2021; Zeng et al., 2022). Therefore, the combination of AI with NGS is expected to further enhance the accuracy of precision medicine for hepatocellular carcinoma.

In addition to identifying response biomarkers by NGS, establishing individual drug screening platforms is an urgent need to overcome drug resistance. Fortunately, the rapid development of bioengineering technology has made it possible to develop excellent HCC preclinical models to track tumor evolution and study drug resistance mechanisms. At present, patient-derived xenograft (PDX) and patient-derived organoid (PDO) models are widely used, which can simulate the occurrence of liver cancer and the tumor microenvironment and provide a preclinical platform for drug screening, biomarker development, drug resistance changes, and mechanism research. Additionally, the rapid development of three dimensional (3D) biology in recent years has provided more possibilities for the development of HCC preclinical models.

## 4 Bioengineering

### 4.1 Patient-derived xenografts

**FIGURE 1:**
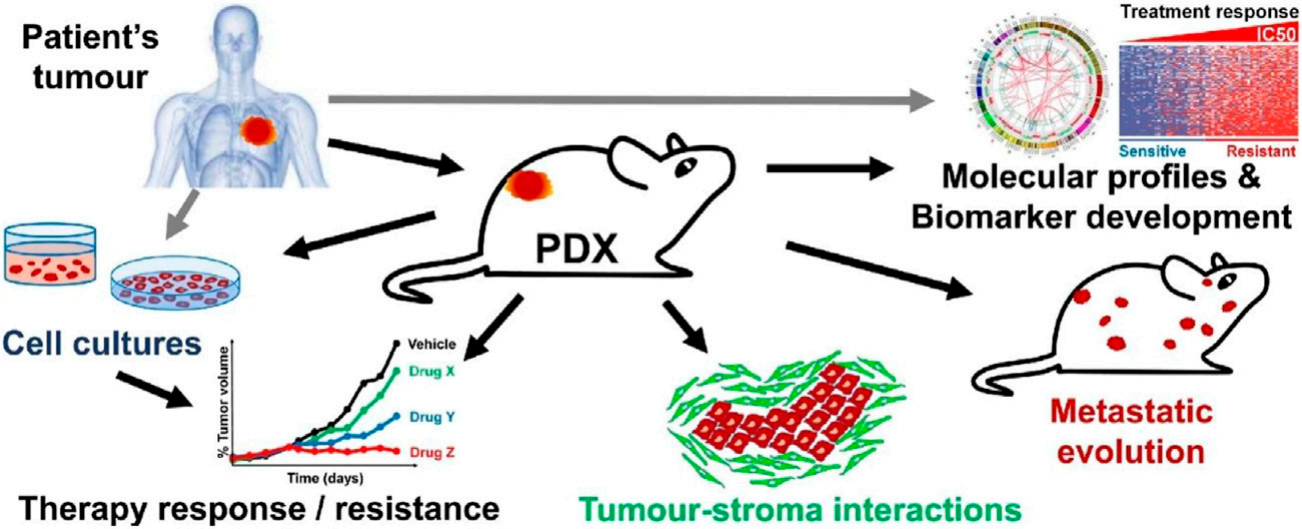
Generation and application of PDX. Patient-derived tumors are implanted in mice, and after the implantation phase, it is transplanted and expanded to generate PDX cohorts suitable for preclinical studies, including drug testing and molecular profiling studies. In addition, PDX-derived tumor samples can be collected to create tissue biobanks, which have important implications for sustainable preclinical research, his figure was cited from Invrea, et al. (2020). Note: This is an open access article distributed under the Creative Commons Attribution License that permits unrestricted use, distribution, and reproduction in any medium, provided the original work is properly cited (CC BY 4.0).

The PDX model first appeared more than 50 years ago and was applied to the research of colorectal cancer (Rygaard and Povlsen, 1969). In 1996, the HCC PDX model was established for the first time (Sun et al., 1996). The subsequent HCC PDX development was slow, cumbersome, and inefficient. However, clinical research results in recent years stimulated hope for the application of PDX models in liver cancer research. The HCC PDX model is currently a mature and ideal tumor model for HCC, which accurately recapitulates the genetic complexity of human tumors, mimics the *in vivo* interactions of tumors with their surrounding tissues and has good clinical predictability (Figure 1); (Brown et al., 2018; Invrea, et al., 2020)

One of the key points in establishing HCC PDX models is the selection of animals for transplantation. Another key point is the selection of the injection site. Subcutaneous injection of cells/tumor (heterotopic model) is the simplest *in vivo* method, which can more accurately measure tumor growth and response to treatment (Brown et al., 2018). However, this approach results in subcutaneously transplanted tumors lacking a tumor-associated microenvironment. Orthotopic transplantation can provide a microenvironment similar to that of the matched tumor tissue and has a rich blood supply, which greatly retains the specificity and microenvironmental characteristics of the patient’s tumor (Hernandez-Gea et al., 2013). It is currently the most ideal transplantation method, but compared with heterotopic transplantation, its technical difficulty, low success rate, cumbersome tumor assessment, and other issues affect its expanded application.

Currently, the most commonly used model is a xenograft model implanting patient-derived samples in an immunodeficient mouse to prevent the mouse’s immune system from rejecting the human tumor. However, this PDX model in immunodeficient mice cannot be used to study immunotherapeutic approaches. Therefore, recapitulating the complexity of the human immune system in preclinical models is critical for studying immunity and immunotherapy in HCC. One strategy to achieve this goal is to use humanized mouse models modified to contain human immune cells (Brown et al., 2018; Zhao et al., 2018). There are different approaches to humanizing the mouse immune system. Even so, a lot of work is needed to fully personalize humanized mice to match each patient sample.

PDXs are already a mature and excellent individualized treatment platform in the preclinical model of HCC, and have shown advantages as a preclinical model in mechanism research, drug and biomarker screening, and combined clinical trials (Hu et al., 2019). PDXliver is the first public database of liver cancer PDX models, including drug response data, which fully reflects the heterogeneity of HCC and is conducive to the discovery of biomarkers for specific treatments (He et al., 2018). Recently, Jin et al. (2021) found that lenvatinib and gefitinib had antitumor activity in HCC PDX with high expression of epidermal growth factor receptor (EGFR), and EGFR has been used as a biomarker to achieve a more informed patient stratification in clinical trials. The HCC PDX model has proven valuable in exploring many different aspects of precision oncology in preclinical research. However, the time commitment, low success rate, and large resource requirement of PDX also limit its wide application.

### 4.2 Patient-derived organoids

To circumvent the limitations of two dimensional (2D), monolayer cell line tumor models, researchers have been trying to grow tumors in 3D for a long time using methods, such as liver slices and mechanical 3D culture devices (Tharehalli et al., 2019). However, these models fall short in terms of culture time and preservation of the original tumor characteristics. One of the main purposes of 3D tumor cell culture is to simulate the growth environment of tumors in the human body and to preserve the genetic and histological characteristics of the parent tumor to the greatest extent. Only such preclinical models can be applied to individualized treatment.

**FIGURE 2:**
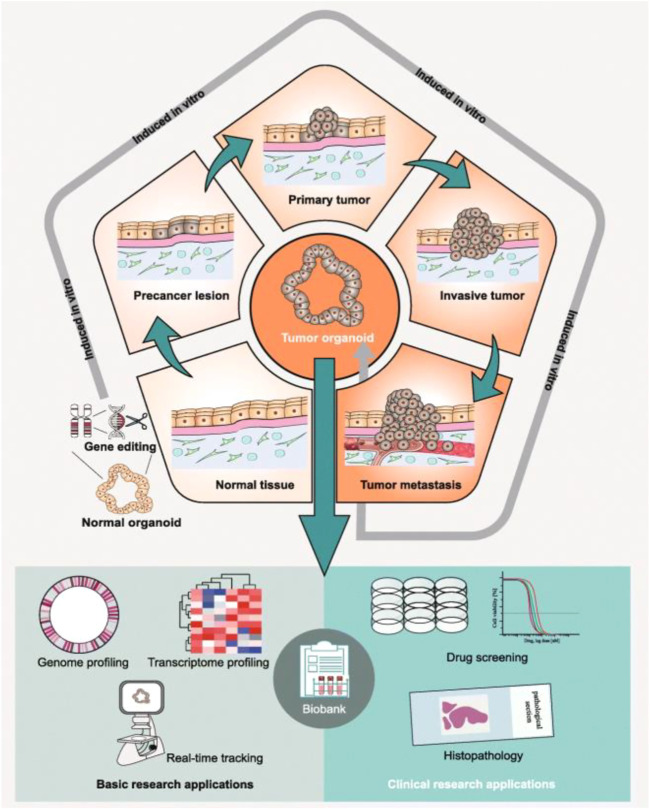
Preclinical applications of patient-derived tumor organoids. Whether in preclinical basic research or clinical research, patient-derived tumor organoids have great application value. This figure was cited from Fan, et al. (2019). Note: This is an open access article distributed under the Creative Commons Attribution License that permits unrestricted use, distribution, and reproduction in any medium, provided the original work is properly cited (CC BY 4.0).

In 2012, the first tumor organoids were developed, derived from intestinal tumors (Sato et al., 2011). Tumor organoids have given rise to a new concept of 3D culture, characterized by self-organized 3D structures that mimic the original *in vivo* structure of an organ or tumor and can be obtained from different sources (Figure 2); (Fan, et al., 2019; Tuveson and Clevers, 2019) Thus far, organoids have been derived from organ-specific adult stem cells, pluripotent stem cells (PSCs), embryonic stem cells, or induced pluripotent stem cells (iPSCs), as well as tumors. Huch et al. (2015) established the first liver organoids from mice and human liver stem cell organoids, in 2013 and 2015, respectively, in which stem cells can be expanded for a long time and differentiated into biliary or hepatic cells according to the composition of the medium. In addition, tissue-specific organoids can be established using PSCs. Takebe et al. (2013) constructed liver organoids from human iPSCs combined with endothelial and mesenchymal cells in Matrigel. At present, liver multicellular co-culture organoids have been reported by many studies, including a mixed culture of hepatocytes and various mesenchymal cells and iPSC-derived liver organoids on perfusion microcolumn chips.

The application of the above healthy liver organoids in tumor therapy is mainly used to the study carcinogenesis, such as the carcinogenesis induced by the hepatitis B virus. Of course, organoids from patient-derived liver tumors are the most direct and effective method to study the individualized treatment of tumors. There are two main sources of PDOs, needle biopsy and surgically obtained human tumor specimens. Due to the difficulty and complexity of organoid culture, both routes are less effective in establishing HCC organoids (37.5% and 26%, respectively) (Broutier et al., 2017; Nuciforo et al., 2018). However, even at low tumor to stromal cell ratios, there are opportunities to establish PDO. This helps avoid short comings of NGS. Furthermore, it is more important that HCC PDOs are highly concordant with original tumor biopsies in terms of growth pattern, degree of differentiation, expression of HCC-specific markers, genomic alterations, and ability to form tumors in xenograft models (Broutier et al., 2017; Nuciforo et al., 2018). This makes PDOs more suitable for precision medicine, including targeted therapy resistance research, drug screening, and treatment response prediction. Li et al. established HCC organoids for drug screening using surgical specimens of primary human liver cancer, and proved that PDOs can be used as preclinical models for the individualized treatment of HCC (Li et al., 2019). By establishing HCC PDOs, Wang et al. (2020); Leung et al. (2020) found reactivation of Hedgehog signaling and receptor tyrosine kinase-induced MEK/ERK and AKT signaling pathways may be related to sorafenib resistance in HCC. In addition to Matrigel encapsulation, a recent study used hydrogel capsules to culture HCC PDOs to simulate the tumor microenvironment of liver cancer, and demonstrated the heterogeneity of the platform for targeted drugs and other applications that can be used to assist individualized therapy (Dong et al., 2022). Clinical trials (NCT05384184 and NCT02436564) examining HCC PDOs are already underway.

The ongoing development of HCC PDOs will be focused on better simulating the tumor microenvironment *in vivo*. Loh et al. (2021) constructed a HCC PDO model and explored signaling axes that enhance hepatocyte resistance to sorafenib by culturing organoids in conditioned medium to mimic the tumor microenvironment. Recently, Lim et al. (2022) used a hydrogel system to establish a co-culture model to mimic and characterize pro-angiogenic secretory signaling between hepatoma cells and endothelial cells *in vitro*. A PDO-TME model combining PDOs and the tumor microenvironment cells will provide a more accurate platform for liver cancer-targeted therapy to avoid heterogeneity and overcome drug resistance research, and it will also be more conducive to the development of new drugs.

### 4.3 3D bioprinting of HCC

In recent years, additive manufacturing, or 3D printing, has been applied to biomedicine, called 3D bioprinting (3DP). The development of 3DP has opened a new chapter in bioengineered medicine (Murphy and Atala, 2014). 3DP is mainly achieved through inkjet, micro-extrusion, or laser-assisted bioprinting, among which micro-extrusion is the most widely used (Mandrycky et al., 2016; Matai et al., 2020). The core material of 3DP is bio-ink and its potential for clinical translation depends on the formulation of the bio-ink (Gu et al., 2022). Bio-inks that have been extensively studied include alginate, fibrinogen, gelatin, collagen, chitosan, agarose, Pluronic, hyaluronic acid, GelMA, PEG, and decellularized extracellular matrix. The choice of bio-ink is also based on the choice of bioprinting technology (Gu et al., 2022).

**FIGURE 3:**
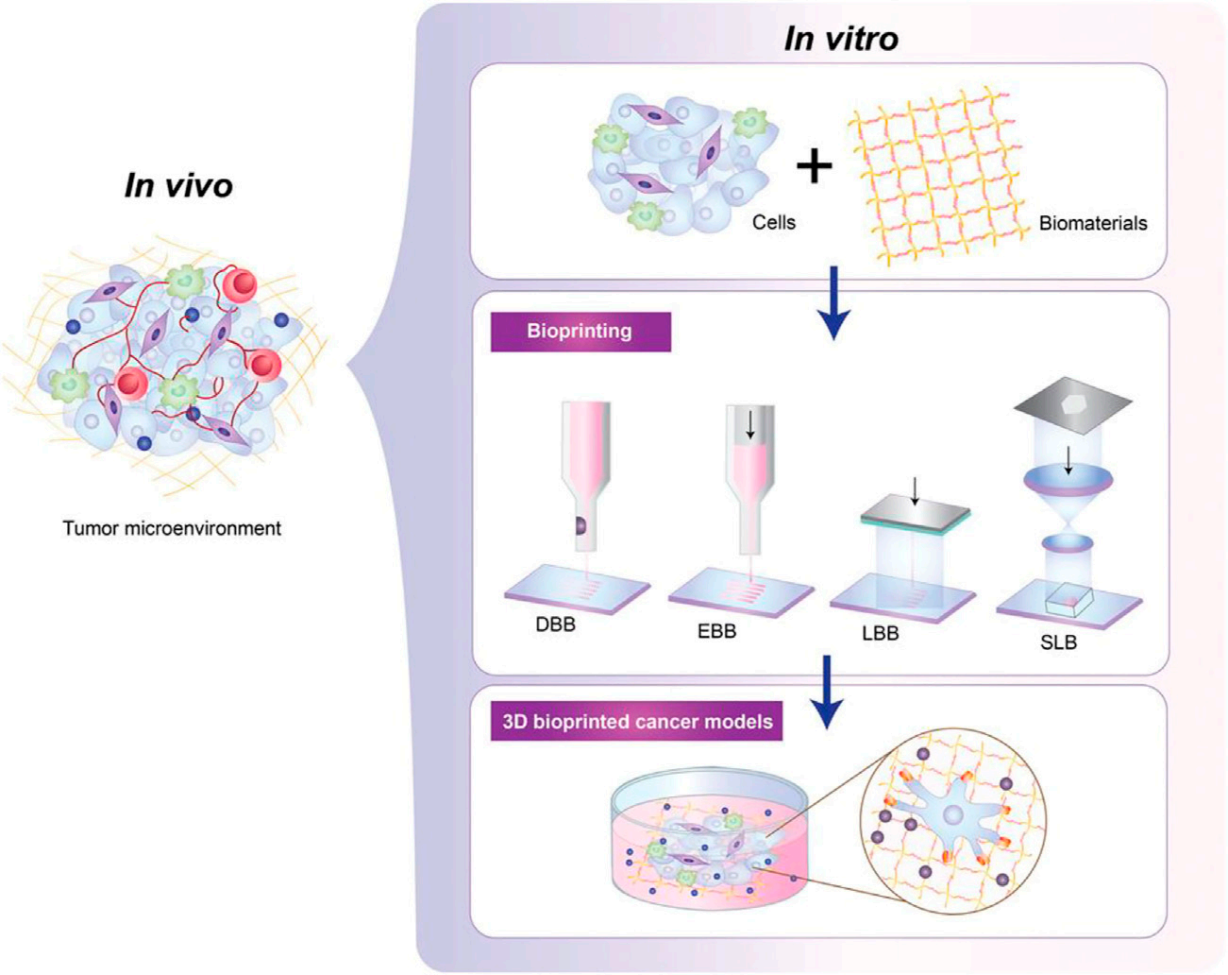
The development process of patient-derived tumor 3DP models. Tumor cells are wrapped in specific bio-inks with high biocompatibility, and then 3DP tumor models can be generated in different ways in high-precision bioprinters, including droplet-based (DBB), extrusion-based (EBB), laser -based (LBB) and stereolithography (SLB) bioprinting. This figure was cited from Jung, et al. (2022). Note: This is an open access article distributed under the Creative Commons Attribution License that permits unrestricted use, distribution, and reproduction in any medium, provided the original work is properly cited (CC BY 4.0).

3DP has been widely used in cancer research (Almela et al., 2018; Wang et al., 2018; Swaminathan et al., 2019; Sbirkov et al., 2021; Xie et al., 2021). 3DP tumor models can provide physiologically relevant cell-cell and cell-matrix interactions by mimicking the 3D heterogeneity of real tumors (Figure 3); (Knowlton et al., 2015; Augustine et al., 2021; Jung, et al., 2022) We initially created a 3DP model of HepG2 cells by 3DP technology based on a gelatin-sodium alginate bio-ink system (Sun et al., 2020). We found that compared with 2D-HepG2 cells, the 3DP-HepG2 model displayed significantly increased levels of various liver function-related proteins and genes as well as those involved in proliferation, metastasis, drug resistance, anti-tumor immunosuppression, and tumor cell epithelial-mesenchymal transition. This gives the 3DP tumor model unique advantages in the preclinical research of liver cancer and the 3DP tumor model can be used as a more suitable platform for anti-tumor drug development. Next, we have previously revealed that patient-derived primary hepatocellular carcinoma cells could maintain high activity long-term in gelatin-sodium alginate bio-ink-based 3DP model and could be used for liver cancer-targeted therapy drug testing for the prediction of personalized therapy (Xie et al., 2021). We found that 3DP tumor models offer advantages in terms of cost, modeling success rate, maintenance of cell viability, establishment success rate, and low time commitment. 3DP printing relies on a computer to generate the design, high precision, and repeatability of the tumor model, which has inherent advantages over the PDX and PDO models. In addition, if a technological breakthrough in the rapid expansion of primary liver cancer cells can be achieved, 3D bioprinting will play a greater role in the application of liver cancer *in vitro* models.

3DP cancer models can recapitulate a tumor’s microstructure and function and preserve the parental tumor’s features. Therefore, it is an ideal 3D, preclinical model with a higher success rate in construction and drug testing than existing traditional cancer models (Table 3); (Shukla et al., 2022) In addition, the absolute advantage of 3DP models as preclinical models is that printers can enable the fabrication of high-resolution microstructures to reproduce the complexity of the tumor microenvironment, including the vascularization of tumors (Shukla et al., 2022). At present, bioprinting has been applied in 3DP models of breast cancer and glioblastoma (Zhou et al., 2016; Heinrich et al., 2019). The application of a 3DP-TME cancer model combining 3DP HCC tumors and the tumor microenvironment needs to be verified by additional research.

**TABLE 3:** Relative advantages related to PDX and PDO.

	PDX	PDO	3DP
Establishment success rate	−	−	++
Cost	−	+	++
Recapitulates tumoural heterogeneity	+	+	+
Multi-Cell culture conditions	+	+	++
Microenvironment construction	++	+	++
High-throughput screening	−	+	+

3DP can also be combined with cancer chip technology to reproduce key tumor microenvironmental characteristics (Monteiro et al., 2022; Shukla et al., 2022) and generate an increasing number of biomimetic tumor models for precision and personalized medicine, which is of great significance for studying HCC drug resistance and tumor evolution *in vitro*.

## 5 Conclusion

The systemic treatment of HCC has made considerable progress, and a higher number of treatment options are now available. However, due to the huge heterogeneity of HCC, the focus of HCC research should not only be on drug development but also on how to accurately select individualized treatment options. This will save more advanced patients from the toxic side effects of drugs with no tumor response and increase patient survival time. The development of bioengineered models of tumors shows great promise for personalized medicine and improved HCC outcomes. At present, there are relatively few clinical studies, and more studies using clinical specimens are needed to clarify whether the bioengineered model of HCC is suitable for capturing intratumoral heterogeneity and predicting patient response to treatments such as targeted therapy.
